# Large-Field-of-View Visualization with Small Blind Spots Utilizing Tilted Micro-Camera Array for Laparoscopic Surgery

**DOI:** 10.3390/mi11050488

**Published:** 2020-05-10

**Authors:** Alex J. Watras, Jae-Jun Kim, Jianwei Ke, Hewei Liu, Jacob A. Greenberg, Charles P. Heise, Yu Hen Hu, Hongrui Jiang

**Affiliations:** 1 Department of Electrical and Computer Engineering, University of Wisconsin-Madison, Madison, WI 53706, USA; watras@wisc.edu (A.J.W.); jkim724@wisc.edu (J.-J.K.); jke9@wisc.edu (J.K.); hliu265@wisc.edu (H.L.); 2 Department of Surgery, University of Wisconsin School of Medicine and Public Health, Madison, WI 53792, USA; greenbergj@surgery.wisc.edu (J.A.G.); heise@surgery.wisc.edu (C.P.H.)

**Keywords:** large field of view, small blind spots, miniaturized cameras, laparoscopy, bean drop task

## Abstract

Existing laparoscopic surgery systems use a single laparoscope to visualize the surgical area with a limited field of view (FoV), necessitating maneuvering the laparoscope to search a target region. In some cases, the laparoscope needs to be moved from one surgical port to another one to detect target organs. These maneuvers would cause longer surgical time and degrade the efficiency of operation. We hypothesize that if an array of cameras can be deployed to provide a stitched video with an expanded FoV and small blind spots, the time required to perform multiple tasks at different sites can be significantly reduced. We developed a micro-camera array that can enlarge the FoV and reduce blind spots between the cameras by optimizing the angle of cameras. The video stream of this micro-camera array was designed to be processed in real-time to provide a stitched video with the expanded FoV. We mounted this micro-camera array to a Fundamentals of Laparoscopic Surgery (FLS) laparoscopic trainer box and designed an experiment to validate the hypothesis above. Surgeons, residents, and a medical student were recruited to perform a modified bean drop task, and the completion time was compared against that measured using a traditional single-camera laparoscope. It was observed that utilizing the micro-camera array, the completion time of the modified bean drop task was 203±55 s while using the laparoscope, the completion time was 245±114 s, with a *p*-value of 0.00097. It is also observed that the benefit of using an FoV-expanded camera array does not diminish for subjects who are more experienced. This test provides convincing evidence and validates the hypothesis that expanded FoV with small blind spots can reduce the operation time for laparoscopic surgical tasks.

## 1. Introduction

Laparoscopic surgery involves the use of a thin, tubular device called a laparoscope that is inserted through a keyhole incision into the abdomen to perform operations that used to require the body cavity to be opened. Because the procedure involves smaller incisions, laparoscopic surgery promises less trauma, shorter hospital stays and faster recoveries [1]. The laparoscope itself is a long, rigid fiber-optic instrument that is inserted into the body to view internal organs and structures. It is equipped with a miniature digital camera mounted at the end of the tube together with a light source. The surgery is guided by the close-up video imaging provided by such a camera and is viewed externally on a monitor.

Laparoscopy is a technically complicated surgery that requires excellent hand-eye coordination and an almost intuitive ability to navigate delicate internal structures. The surgeon must also rely on a patient-side assistant to position the camera correctly. Furthermore, the limited field of view (FoV) afforded by a single-camera laparoscope makes it difficult to navigate inside the body cavity to reach the targeted surgical region, or to monitor the surrounding anatomy as in an open-cavity surgery.

Several improvements to the current design of the single-camera laparoscope have been proposed. Most of these proposals focus on replacing the single-camera assembly with a pair of binocular cameras to provide 3D vision [2,3,4]. Bhayani et al. [5] and Tanagho et al. [6] reported human test results that found that a 3D visualization system makes it easier to perform a standardized laparoscopic task than using a conventional 2D visualization system. Recently, several commercial 3D laparoscopy products have been on the markets and quite a few test results have been reported comparing the effectiveness of 3D versus 2D laparoscopic surgeries [7,8,9,10,11,12]. Some of these systems are robot-assisted [9,13]. While some wide-FoV endoscopes can reach 100–170${}^{\circ }$ FoV, they are prone to undesirable distortion [14], and may require image processing techniques to remove the distortion before the video footage can be used.

A second direction for improving the laparoscope is to incorporate multiple (more than two) cameras to enlarge the FoV. The MARVEL system [15,16,17] uses multiple wirelessly controlled pan/tilt/zoom camera modules to facilitate intra-abdominal visualization. In the MARVEL system, cameras need to be individually inserted through separate incisions (other than surgical ports) into the abdomen and pinned via needles to the inside of the abdominal wall. Anderson et al. proposed a VTEI system [18,19] with a static micro-camera array that still needs to be surgically inserted into the abdomen and attached directly to the abdomen wall. Both the MARVEL and the VTEI systems intend to provide an enlarged FoV comparable to that of an open-cavity surgery. In addition to the MARVEL and the VTEI systems, a multi-view visualization system was developed to enlarge FoV by using two miniaturized cameras and a conventional endoscope [20]. This visualization system reduced both the procedure time and the number of commands for operating the robotic endoscope holder. However, the proposed visualization system can only enlarge the FoV in one direction due to the limitation in camera placement and was not able to combine the multiple images into a single large one, which could degrade the efficiency of operation.

**Figure 1:**
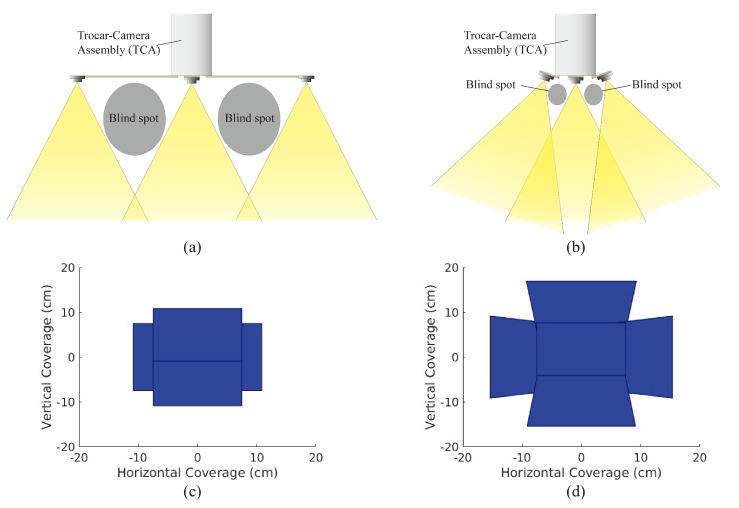
Field of View (FoV) Coverage: Careful choice of camera placement can reduce blind spots (**a**,**b**) while increasing total FoV (**c**,**d**) at the bottom of the trainer box (depth = 16.5 cm).

To alleviate the limited FoV and low efficiency of operation in traditional laparoscopic visualization, previously, we demonstrated a trocar-camera assembly (TCA) [21]. However, the large spacing between the miniaturized cameras causes large blind spots and limits the operation of TCA in case of impacting the abdominal wall (Figure 1a). Here, we demonstrate an improved design of TCA that has a tilted micro-camera array to mitigate the blind-spot issues of the previous version of TCA. In Figure 1b, it is shown that by properly tilting the orientation of the cameras in this new design, the blind spots and spacing between cameras can be significantly reduced.

Changing the camera array from a 75 mm arm length with 0${}^{\circ }$ camera rotation to an 18 mm arm length with 20${}^{\circ }$ camera rotation raises the depth at which camera overlap begins to occur from 13 cm to 10 cm, thus allowing for significantly more usable scene depth and thus less blind spots. Furthermore, as can be seen in Figure 1c,d, we can generate a 2D FoV by intersecting the viewing frustum of each camera in the array with the bottom of the FLS trainer box (represented by a plane at a depth of 16.5 cm). As the camera array does not have the same rectangular FoV frustum that single-camera systems have, the traditional vertical and horizontal solid angles used to measure FoV do not apply. Instead, the area of this 2D FoV serves as an equivalent measure.

This micro-camera array that uses tilted cameras provides larger FoV compared to that accomplished via non-tilted cameras (Figure 1c,d). For deployment of the micro-camera array, this new design of TCA can utilize the same mechanical system demonstrated in Reference [21] except the foldable camera support that consists of mechanical arms, a camera holder, and torsion springs. Among the components of the foldable camera support, the torsion springs need to be redesigned to achieve the desired tilting angle of the cameras because the initial angle of the torsion springs determines the tilting angle of the cameras. Once a surgical port is inserted into the patient’s abdominal cavity, the miniaturized cameras are flared out from the in vivo end of the port by a specially designed mechanical system that consists of several micro-machined components. As such, the surgical port still allows the passage of other surgical instruments after camera deployment. This advantage of TCA can still be maintained in this new design. Since the abdomen is inflated with ${\mathrm{CO}}_{2}$ during the operation, and the ports are on the anterior abdominal wall, there is ample working space within the abdominal cavity to deploy the miniaturized cameras. After the completion of the operation, the mechanical system that holds the miniaturized cameras are folded back inside the port and the whole trocar including the micro-camera array is retrieved.

In this work, we hypothesize that if an array of cameras can be deployed to provide a stitched video with an expanded FoV and small blind spots, the time required to perform multiple operations at different spots can be significantly reduced. While this seems to be intuitively correct, to the best of our knowledge, no empirical study has been performed to validate this hypothesis.

**Figure 2:**
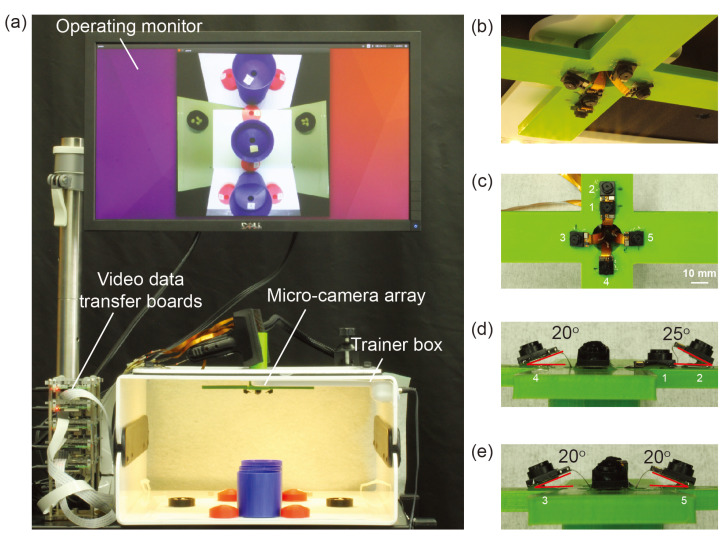
Micro-camera Array Visualization System: (**a**) The micro-camera array is inserted into the trainer box. The operating monitor shows the stitched video that used video data from the micro-camera array transferred through the video data transfer boards. (**b**) The miniaturized cameras are placed on a 3D printed scaffold to allow for the use of the visualization software during testing. The camera position has been chosen to maximize the coverage of the bottom of the trainer box while minimizing parallax artifacts in the visualization. (**c**) Front view of the micro-camera array with numbers indicating camera position. (**d**,**e**) Side view of the micro-camera array with numbers indicating camera position and tilting angles.

To validate this hypothesis, we conducted an experiment that compares the time taken to complete a laparoscopic surgical training task using a proof-of-concept micro-camera array against that of using a conventional single-camera laparoscope. We utilized a simplified version of the TCA which contained cameras with optimized tilting angles but without the deployment mechanism (Figure 2) [21]. Instead, this micro-camera array was permanently installed on a laparoscopy surgery trainer box. We focus on investigating the new camera placement and the validation of the above hypothesis with surgeons, rather than repeating the work on the mechanical deployment system previously developed and reported in Reference [21]. The video streams of the micro-cameras were transmitted to an outside computer server where they were stitched to form a video mosaic stream with an expanded FoV.

## 2. Materials and Methods

### 2.1. Micro-Camera Array Visualization System

**Figure 3:**
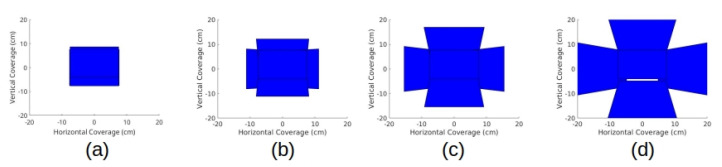
FoV Selection: A comparison of the FoV for cameras placed at a (**a**) 0${}^{\circ }$, (**b**) 10${}^{\circ }$, (**c**) 20${}^{\circ }$, and (**d**) 30${}^{\circ }$ angle. When the angle is too small, the FoV is not maximized. On the contrary, when the angle is too large, holes begin to form in the FoV.

The micro-camera array visualization system consisted of a micro-camera array, a trainer box, video data transfer boards, operating monitor, and a laptop implementing a video stitching algorithm (Figure 2a). We used Raspberry Pi 3 model-B boards (Adafruit, New York, NY, USA) as video data transfer boards. Five miniaturized cameras (Raspberry Pi MINI camera module (FD5647-500WPX-V2.0), RoarKit, AliExpress, China) were used to form a micro-camera array. The cameras were attached to an in-house designed, 3D printed socket to mount the micro-camera array in the trainer box (Figure 2b). We placed four cameras about 18 mm and one camera about 28 mm away from the center of the port (Figure 2c). The non-tilted camera 1 in Figure 2c provided a central view of the field in the trainer box and the four tilted cameras (2 to 5) provided four different side views. To enlarge the FoV and reduce blind spots generated by a lack of detection overlap between cameras, we tilted the four cameras (2 to 5) based on the angles calculated using an algorithm we developed earlier [22]. To find the optimized angle of the cameras, we calculated the FoV of the micro-camera array as a function of the tilted angle of the cameras (Figure 3) and found that the optimized angle of the cameras is 20${}^{\circ }$. A tilted angle of 10${}^{\circ }$ cannot maximize the FoV and holes begin to form in the FoV when the tilted angle is 30${}^{\circ }$. To minimize the effect of parallax on the visualization, we reduced the allowable arm length to the minimum possible. As noted in our previous work [22], the total FoV increases as the camera angle increases. Thus we chose to place cameras such that these angles are maximized while maintaining sufficient overlap to satisfy the stitching constraints. Camera 1 was placed at a 0-degree angle to the micro-camera array (Figure 2d). Cameras 3, 4, and 5 were placed at an angle of 20${}^{\circ }$ to the micro-camera array (Figure 2d,e). Camera 2, being closer to camera 1 than the others, was placed at an angle of 25${}^{\circ }$ to the micro-camera array (Figure 2d). The tilted angle of camera 2 was slightly larger than the other cameras and was slightly off the target angle 20${}^{\circ }$. It helped to enlarge the FoV without forming holes in the FoV because camera 2 is closer to camera 1 than the other cameras.

**Figure 4:**
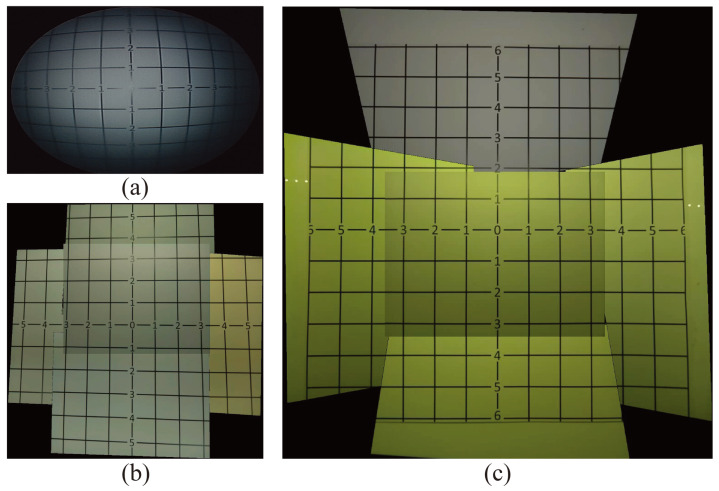
FoV Measurement: Captured images from (**a**) a traditional laparoscope, (**b**) a non-tilted camera array, and (**c**) the micro-camera array with tilted cameras for FoV measurement using grid lines. The micro-camera array can provide larger FoV and detect larger area (133 visible squares) compared with the traditional laparoscope (56) or non-tilted array (84).

To validate the effectiveness of this proposed micro-camera array on the laparoscopic surgery, we mounted it on to an FLS laparoscope trainer box (to be discussed a bit later) and compared the FoV of the stitched video frame against the FoV of a single-camera visualization system and a non-tilted camera array. For comparison, we showed the images of the same grid paper under these three visualization systems in Figure 4. Clearly, the number of square grids observable by the traditional visualization system (Figure 4a) and the non-tilted camera array (Figure 4b) is 56 and 84 squares, respectively, which was much lower than the 133 squares visible in the stitched image of the micro-camera array visualization system (Figure 4c). This represents that the visible area using the micro-camera array visualization system increased by 137.5% and 58.3%, compared with the traditional laparoscope and non-tilted camera array, respectively. In addition, the FoV of the traditional laparoscope visualization was 62${}^{\circ }$, and the FoV of the micro-camera array was 85${}^{\circ }$, respectively. that the FoV of the micro-camera array increased by 37.1% and 14.9%, compared with the traditional laparoscope and non-tilted camera array, respectively.

In this work, we succeeded in achieving larger FoV with fewer blind spots than in the previous work that used non-tilted cameras [21]. Furthermore, this design of the micro-camera array was able to reduce the space wasted by camera placement in the surgical field. Furthermore, the distance between the center of the camera and the center of the port was significantly reduced (see Figure 2c) compared to the previous work (the distance was 75 mm in Reference [21]). The new distances are 28 mm for camera 2 and 18 mm for the other cameras.

### 2.2. Video Stitching Algorithm

A real-time multi-view video stitching algorithm was developed to process video feeds from the five cameras to form a video mosaic whose FoV is the union of FoVs of all five cameras. At this early stage of development, we designated the non-tilted center camera as the default viewpoint (the main view) and augmented its FoV with the four peripheral cameras (the side views) using a stitching algorithm similar to the one proposed by Zheng et al. [23]. At the beginning of the operation, intrinsic camera parameters and extrinsic camera poses of the five cameras were estimated through a camera calibration process. Since the camera array remains stationary through the operation, the calibration needs to be performed only once initially.

For each video frame, the FoV of the stitched video frame was the FoV of the main view (Camera 1) augmented by FoVs of the four side views (Cameras 2–5). For each side-view camera, the FoV of each side-view was geometrically transformed to align it to Camera 1 through a homography $H_{i}$, and then the two are combined using a blending mask $M_{i}$. Both $H_{i}$ and $M_{i}$, i=2,3,4,5 were estimated during the initial calibration and then used throughout the operation.

The video stitching algorithm was implemented on a Linux laptop that had an Intel Xeon E3-150M v5 processor with 4 cores, and 32 GB of main memory. Multi-threading was leveraged to accelerate the computation. At the time of the experiment, this implementation of the video stitching algorithm achieved a processing rate of 25 stitched video frames per second.

### 2.3. Modified Bean Drop Task

The bean drop task was designed to emulate the laparoscopic surgical task of moving from one location to another during surgery [24]. In a usual bean drop task, a subject is asked to pick up beans from a saucer using a grasper and transfer them one by one to a cup on the other side of the trainer box. In our experiment, we devised a modified bean drop task with increased complexity. We hypothesized that such modification would mitigate the performance (overall completion time) discrepancies due to different skill levels of subjects.

**Figure 5:**
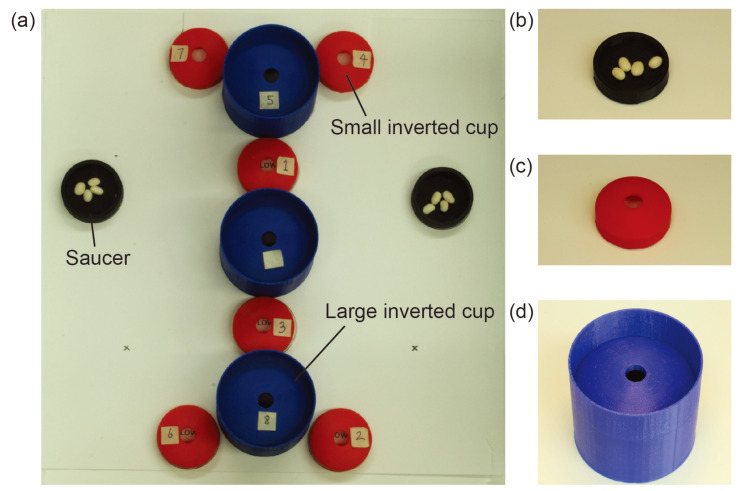
Modified Bean Drop: Our modified bean drop task consisted of 2 saucers with a diameter of 40 mm (**b**), 6 small inverted cups with diameters of 40 mm (**c**), and 3 large inverted cups with diameters of 62 mm (**d**), all placed in the pattern shown in (**a**). This pattern was designed to be large enough that it cannot be fully seen with the traditional laparoscope to emphasize the increased FoV offered by the micro-camera array. The traditional laparoscope needs to survey the scene being able to complete the task and occluding elements made the survey difficult.

In the modified bean drop task, a subject was asked to move eight separate beans, each to a separate location in the trainer box. In addition, two types of cups as shown in Figure 5b,c were used. These cups were placed in a pattern shown in Figure 5a, such that the taller cups may obscure the smaller cups if viewed from the wrong angle. The pattern was symmetric so that completion time would be independent of handedness. We implemented these changes to require the subject to exercise a level of strategic planning and situational awareness.

We laid out a set of nine inverted cups and two saucers in the pattern seen in Figure 5. Eight of the nine inverted cups were labeled with the numbers 1–8, while the remaining inverted cup was left unlabeled to serve as an obstacle hindering the visual search process. These labels allowed us to randomize the order of the individual bean drop tasks to introduce yet more planning. In each of the two saucers, we placed four beans. By limiting the number of beans available to our subjects, we once again emphasized the importance of task planning.

Two surgical graspers are provided to perform the modified bean drop task so that the subject may use both hands to perform the task. During the transfer of a bean from a saucer to a cup, it may be dropped accidentally. The subject may pick up a dropped bean to complete the task with some timing penalty. However, if the subject cannot see the dropped bean via the visualization system or cannot reach it via the provided ports, that bean is deemed lost during transfer. When a bean is lost, the protocol requires the subject to move on to transfer the next bean to the required numbered cup. The completion time for each of the four tasks and the number of beans dropped during each task would be recorded for subsequent data analysis.

### 2.4. Laparoscope Trainer Box

A standard FLS laparoscopic trainer box designed for practicing laparoscopic procedures was used to validate and evaluate the effectiveness of the proposed micro-camera array. The trainer box is a low fidelity form of simulation but has known validity evidence for laparoscopic surgeries [25]. By default, the box contains two incisions for inserting instruments and a built-in camera placed on the inside of the box in between the instrument incisions.

**Figure 6:**
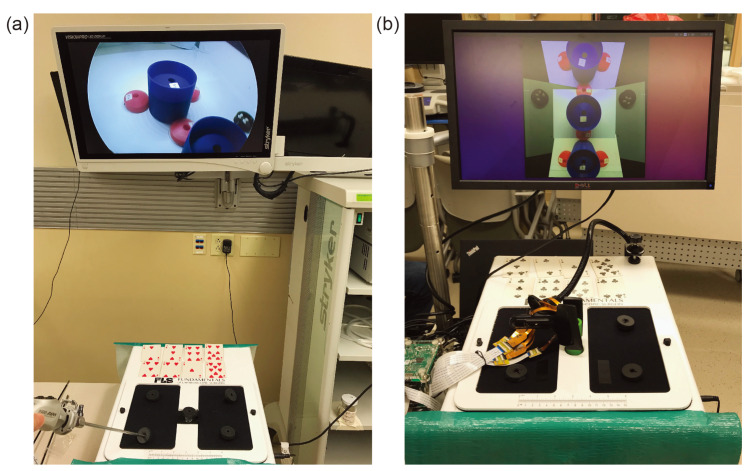
Test Environment: A subject attempted to complete a modified bean drop task in both the traditional laparoscopy visualization environment (**a**), and the micro-camera array visualization environment (**b**). The new environment freed up a hand so that the surgeon can utilize multiple grasping instruments without needing an assistant to hold the camera.

As shown in Figure 6, in this experiment, the built-in camera is removed to make room for the micro-camera array and extra incisions were added. The micro-camera array is mounted through the central incision to provide a central viewpoint with a stitched FoV covering the entire bottom of the trainer box. A clamp was applied outside of the box to ensure that the array stayed fixed in its location. Other incisions were placed on top of the trainer box so that the surgeon could easily view or reach any location at the bottom of the trainer box. As shown in Figure 5, several cups and saucers were affixed to the bottom of the trainer box using Velcro. These cups and saucers facilitate a modified bean-drop task to be performed in this experiment. The final result of this can be seen in Figure 6.

### 2.5. Subjects and Test Protocol

We recruited 26 test subjects consisting of a medical student, surgical residents, and faculty of the University Hospital of the University of Wisconsin. The group consisted of 1 medical student, 3 first-year residents, 5 second-year residents, 6 third-year residents, 2 fourth-year residents, 3 fifth-year residents, and 6 faculty surgeons. The subjects were randomly recruited based on availability. The medical student had no prior experience with laparoscopic surgery. The first-year residents had less than a year of experience while the other residents had experience approximately equal to their years of residency. The faculty surgeons had extensive prior laparoscopic experience and were recruited based on the fact that they routinely perform minimally invasive (laparoscopic) surgery.

Testing took place in the laparoscopic trainer room of the Simulation Center in the University Hospital over the course of 5 days. During testing, subjects were asked to stop by the testing facility at their leisure to perform a short laparoscopic training task to help test out a new visualization system. There was no restriction on how many subjects were allowed in the testing room, nor were subjects prohibited from watching other subjects perform the task, although they were discouraged from doing so.

When a subject arrived at the testing facility, the subject was given a brief explanation of the difference between the two visualization systems and then given a description of the tasks to be completed. The subject was given a picture similar to Figure 5a but with the numbers removed. The subject did not have access to this picture during the experiment. The subject was told that cards numbered 1–8 would be dealt out in front of them in a random order, that the completion of the task would be timed, and that the task should be attempted to complete as quickly as possible. No strategic advice was given to the surgeon. At no point was any instruction given to the subject as to which ports should be used, or how the camera systems or how the surgical graspers should be manipulated. The subject was allowed to use one or both hands to manipulate the graspers.

After the explanation, the subject was directed to one of the two trainer boxes to begin the task. Each setup as seen in Figure 6 consisted of one visualization system, two surgical graspers, a video display, a set of 8 playing cards, and an FLS trainer box. The cups for the modified bean drop task and 8 white beans were located inside each of the trainer boxes as seen in Figure 5. The test giver made sure that the subject was comfortable with their understanding of the task and ready to begin. After attaining verbal confirmation of the subject’s readiness, the test giver gave the subject a command to begin the task. The subject would then attempt to use the provided visualization system to pick up the beans from the black saucers and place them in the numbered cups in the order dictated by the dealt cards. The test giver proceeded to watch as the subject performed the task. The time to complete was measured manually by the test giver with a stopwatch who gave the subject a command to start and stopped timing when the subject placed the last bean or stated verbally that they were done with the task. The overall completion time was chosen to be the difference between the ending and the starting time readings. At the end, the number of dropped beans was also counted and recorded.

Each subject repeated the above procedure four times during the experiment using the two training boxes: twice with the traditional single-camera visualization system and twice with the proposed micro-camera array visualization system. The experiment protocol did not allow a subject to practice on the modified bean drop task in advance. To mitigate the concern that the subject will perform better in later trials (the third or the fourth) due to familiarity with the modified bean drop task in earlier trials, we chose to have the subject switch visualization systems after the first and third attempts. Specifically, the first and fourth attempts were performed with one visualization system and the second and third attempts were performed with the other. Furthermore, either type of visualization system was chosen alternatively for the first trial.

## 3. Results

The outcome of each modified bean drop task was the total completion time for completing the task. However, not all subjects completed all eight required sub-tasks. Sometimes a bean was dropped prematurely outside the destination cup and there was no way to recover it. When this happened, the protocol required the subject to abort the current sub-task and continue to the next one. As such, the completion time with aborted sub-tasks was shorter than if all eight sub-tasks had been completed successfully. Using the traditional laparoscope, there were a total of 26 aborted sub-tasks. Using the micro-camera array, the number of aborted subtasks increased to 45. Note that the total number of sub-tasks performed was 8×4×26=832.

To rectify the reduction of the completion time due to aborted bean drop, a penalty of 28 s was added to the measured completion time for each occurrence of a failed sub-task. The 28 s penalty was the averaged single sub-task completion time of all subjects for both treatments.

**Figure 7:**
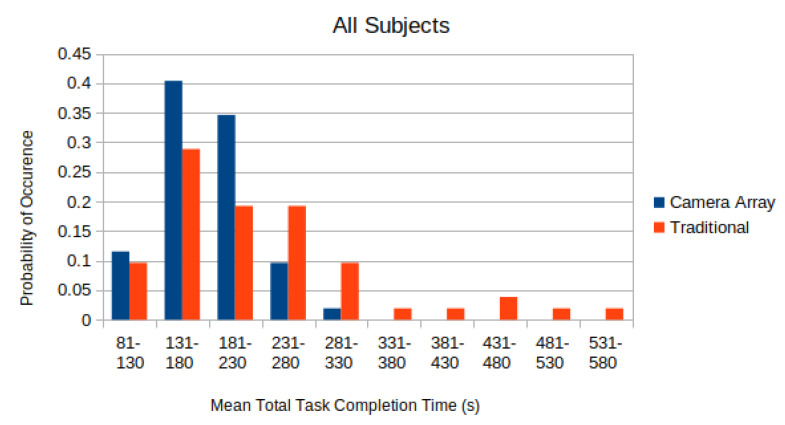
Distribution of Completion Time (with 28 s penalty for each failed sub-task).

In Figure 7, the distributions of the completion time of all test subjects, with 28 s penalty added per aborted sub-task, are depicted. The distribution was normalized so that the sum of all elements equals to unity.

A two-sided paired T-test was applied to each of these distributions in Figure 7 to compare whether the difference in the completion time using the micro-camera array versus the traditional laparoscope was statistically significant. The mean adjusted time-to-complete for the traditional laparoscope was found to be 245±114 s. The mean adjusted time-to-complete on the micro-camera array was found to be 203±55 s. Applying a paired T-test to the data found that subjects completed the tasks faster with the micro-camera array than with the traditional laparoscope (p=0.00097).

Camera hopping is the act of removing and reinserting a laparoscope through the trocar to provide a better viewing angle during the procedure. In the modified bean-dropping test, we recorded multiple camera hopping operations when the laparoscope was used. There was no camera hopping when the micro-camera array was used. Manual inspection of the recorded videos found that on average subjects performed the camera hopping motion 8.28±5.32 times per test.

**Figure 8:**
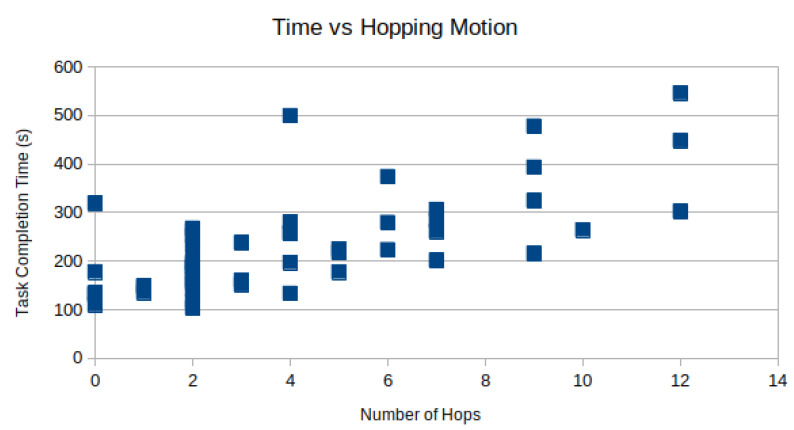
Effect of Camera Hopping: In this chart, we can see that usage of camera hopping has a moderate positive correlation with time-to-complete. (R = 0.69).

However, the time spent moving the camera can not be spent performing the task. In Figure 8, the task completion time is plotted against the number of camera hops for each subject as a scatter plot. As expected, there is a positive correlation between the number of hops and the total time-to-complete. Using linear regression, R = 0.69 is observed.

## 4. Discussion

### 4.1. Validation: Benefit of Larger Field of View

From Figure 7, it is clear that the completion time using the micro-camera array is shorter than that using the traditional laparoscope. Using a significance level of α=0.05, the null hypothesis that states the distribution of the completion time difference using the micro-camera array and the traditional laparoscope has zero mean clearly should be rejected. Hence, this experiment result validates the hypothesis that larger FoV afforded by the micro-camera array can significantly reduce the completion time when performing a simulated laparoscopic surgery (the modified bean dropping test).

Moreover, the completion time utilizing the micro-camera array consistently has a smaller standard deviation than that using the traditional laparoscope. This is also shown in Figure 7 where the distribution of the laparoscope completion time has longer tails leading to much larger standard deviations.

### 4.2. Impact of Subjects’ Experience Using the Traditional Laparoscope

The subjects recruited in this experiment are all familiar with using the laparoscope trainer box except the medical school student. All subjects are unfamiliar with using the micro-camera array viewing system. This familiarity and prior experiences with the traditional laparoscope may contribute to shorter completion time.

### 4.3. Number of Aborted Sub-Tasks

There were a total of 71 aborted sub-tasks out of a total of 832 performed by all 26 subjects. The abort rate was approximately 8.5%. The number of aborted sub-tasks decreased as the experience increased. The abort rate for the micro-camera array was almost twice of the laparoscope. This might be due to the fact that the subjects had variant experience with, and thus were more familiar with the traditional laparoscope, while they were completely new to and were unfamiliar with this new type of micro-camera array visualization system.

### 4.4. Using Both Hands for Operation Using Micro-Camera Array

We observed that with the micro-camera array visualization system, some more experienced subjects used both hands to hold an additional surgical instrument to improve the overall efficiency of completing the modified bean drop task. This illustrates an important benefit of the micro-camera array visualization system. Namely, there will be no need to occupy a dedicated port for inserting the laparoscope and there will be no need to have an assistant to hold the laparoscope during the procedure.

### 4.5. Micro-Camera Array Design

In the proof-of-concept design in this experiment, the cameras were chosen based on their small form factor to fit into the inside of a surgical trocar. The image quality of individual micro-cameras is inferior to that produced by a traditional single-camera laparoscope. Since the micro-camera array is hanging high, it provides a bird-eye view, rather than a close-up view as does the traditional laparoscope. In the next phase of this project, we plan to develop heterogeneous camera arrays that can simultaneously provide a global bird-eye view to facilitate navigation and close-up view to facilitate delicate surgical operations.

In the interest of real-time video stitching, the video stitching algorithm does not compensate for the severe parallax distortion in the stitched video. The image of a laparoscopic surgical instrument may show discontinuity across the boundary between adjacent camera views when the surgical instrument is moving during the experiment. The default viewpoint is also fixed to the top-down view of the center camera. Hence, the subjects are unable to view the scene from an angle to acquire depth perception. In the following development, a multi-planar image stitching algorithm will be developed to mitigate the parallax anomaly. An any-view 3D dynamic rendering viewing system will also be implemented so that surgeons may shift their viewing angle from head-mounted augmented reality goggles to examine the surgical spots from an optimal angle.

## 5. Conclusions

In this paper, an early performance evaluation of a prototype micro-camera array for laparoscopic surgery is reported. We conducted an experiment to validate a hypothesis that larger-FoV visualization can improve the efficiency of navigating laparoscopic surgical tools. In this experiment, human subjects including surgeons, residents, and a medical student were recruited to perform a modified bean drop task using laparoscopic trainer boxes equipped with the micro-camera array and a traditional laparoscopic camera, respectively. The time to complete each task was recorded and analyzed. We observed statistically significant performance improvement in terms of shorter time of completion. Future works will be on developing a laboratory prototype based on this proof-of-concept micro-camera array and corresponding computer vision algorithms to enhance its performance and usability.

## References

[1] Mack M.J. (2001). Minimally invasive and robotic surgery. J. Am. Med. Assoc..

[2] Fuchs H., Livingston M.A., Raskar R., Keller K., Crawford J.R., Rademacher P., Drake S.H., Meyer A.A. (1998). Augmented reality visualization for laparoscopic surgery. Proceedings of the International Conference on Medical Image Computing and Computer-Assisted Intervention.

[3] Hu T., Allen P.K., Nadkarni T., Hogle N.J., Fowler D.L. Insertable stereoscopic 3D surgical imaging device with pan and tilt. Proceedings of the 2008 2nd IEEE RAS & EMBS International Conference on Biomedical Robotics and Biomechatronics.

[4] Hu T., Allen P.K., Hogle N.J., Fowler D.L. (2009). Insertable surgical imaging device with pan, tilt, zoom, and lighting. Int. J. Robot. Res..

[5] Bhayani S.B., Andriole G.L. (2005). Three-dimensional (3D) vision: Does it improve laparoscopic skills? An assessment of a 3D head-mounted visualization system. Rev. Urol..

[6] Tanagho Y.S., Andriole G.L., Paradis A.G., Madison K.M., Sandhu G.S., Varela J.E., Benway B.M. (2012). 2D versus 3D visualization: Impact on laparoscopic proficiency using the fundamentals of laparoscopic surgery skill set. J. Laparoendosc. Adv. Surg. Tech..

[7] Agrusa A., Di Buono G., Buscemi S., Cucinella G., Romano G., Gulotta G. (2018). 3D laparoscopic surgery: A prospective clinical trial. Oncotarget.

[8] Sinha R., Sundaram M., Raje S., Rao G., Sinha M., Sinha R. (2013). 3D laparoscopy: Technique and initial experience in 451 cases. Gynecol. Surg..

[9] Tuschy B., Berlit S., Brade J., Sütterlin M., Hornemann A. (2014). Solo surgery—Early results of robot-assisted three-dimensional laparoscopic hysterectomy. Minim. Invasive Ther. Allied Technol..

[10] Schoenthaler M., Schnell D., Wilhelm K., Schlager D., Adams F., Hein S., Wetterauer U., Miernik A. (2016). Stereoscopic (3D) versus monoscopic (2D) laparoscopy: Comparative study of performance using advanced HD optical systems in a surgical simulator model. World J. Urol..

[11] Berlit S., Hornemann A., Sütterlin M., Weiss C., Tuschy B. (2017). Laparoscopic hysterectomy in the overweight and obese: Does 3D imaging make a change?. Arch. Gynecol. Obstet..

[12] Baum S., Sillem M., Ney J.T., Baum A., Friedrich M., Radosa J., Kramer K.M., Gronwald B., Gottschling S., Solomayer E.F. (2017). What are the advantages of 3D cameras in gynaecological laparoscopy?. Geburtshilfe Und Frauenheilkd..

[13] Da Vinci Surgery | Robotic Assisted Surgery for Patients. http://www.davincisurgery.com/.

[14] Gadermayr M., Liedlgruber M., Uhl A., Vecsei A. (2013). Evaluation of different distortion correction methods and interpolation techniques for an automated classification of celiac disease. Comput. Methods Programs Biomed..

[15] Alqassis A., Castro C.A., Smith S., Ketterl T., Sun Y., Savage P.P., Gitlin R.D. A wireless robotic video laparo-endoscope for minimal invasive surgery. Proceedings of the IEEE Workshop on Robot Vision.

[16] Castro C.A., Smith S., Alqassis A., Ketterl T., Yu S., Ross S., Rosemurgy A., Savage P.P., Gitlin R.D. MARVEL: A wireless miniature anchored robotic videoscope for expedited laparoscopy. Proceedings of the IEEE International Conference on Robotics and Automation.

[17] Castro C.A., Alqassis A., Smith S., Ketterl T., Yu S., Ross S., Rosemurgy A., Savage P.P., Gitlin R.D. (2013). A wireless robot for networked laparoscopy. IEEE Trans. Biomed. Eng..

[18] Anderson A., Lin B., Sun Y. (2013). Virtually transparent epidermal imagery (VTEI): On new approaches to in vivo wireless high-definition video and image processing. IEEE Trans. Biomed. Circuits Syst..

[19] Sun Y., Anderson A., Castro C., Lin B., Gitlin R., Ross S., Rosemurgy A. Virtually transparent epidermal imagery for laparo-endoscopic single-site surgery. Proceedings of the Annual International Conference of the IEEE Engineering in Medicine and Biology Society, EMBS.

[20] Tamadazte B., Agustinos A., Cinquin P., Fiard G., Voros S. (2014). Multi-view vision system for laparoscopy surgery. Int. J. Comput. Assist. Radiol. Surg..

[21] Kim J.J., Watras A., Liu H., Zeng Z., Greenberg J., Heise C., Hu Y.H., Jiang H. (2018). Large-Field-of-View Visualization Utilizing Multiple Miniaturized Cameras for Laparoscopic Surgery. Micromachines.

[22] Watras A.J., Kim J.J., Liu H., Hu Y.H., Jiang H. (2018). Optimal Camera Pose and Placement Configuration for Maximum Field-of-View Video Stitching. Sensors.

[23] Zheng M., Chen X., Guo L. (2008). Stitching video from webcams. Advances in Visual Computing.

[24] Tinelli A. (2011). Laparoscopic Entry: Traditional Methods. New Insights and Novel Approaches Edited.

[25] Diesen D.L., Erhunmwunsee L., Bennett K.M. (2011). Effectiveness of laparoscopic computer simulator versus usage of box trainer for endoscopic surgery training of novices. J. Surg. Educ..

